# Supplementary material for: Nonmuscle Myosin-2B Regulates Apical Cortical Mechanics, ZO-1 Dynamics and Cell Size in MDCK Epithelial Cells
Source: Cells. 2025 Jul 23;14(15):1138. doi: 10.3390/cells14151138 (PMC12346661; doi:10.3390/cells14151138)
Supplement: Supplementary file 1 [file cells-14-01138-s001.zip › Supplementary Table S1 Resources table.pdf]

Supplementary Table S1: Resources table

Reagent of resources	Source	Identifier
**Antibodies**		
Mouse IgG2b monoclonal anti-γ-actin (IB, IF)	Prof. C. Chaponnier, University of Geneva [48]	2A368E2 RRID: AB_2571583
Mouse IgG1 monoclonal anti-β-actin (IB, IF)	Prof. C. Chaponnier, University of Geneva [48]	4C259H12 RRID: AB_2571580
Rat polyclonal anti-PLEKHA6 (IF)	Citilab [79]	RtSZR127
Rabbit polyclonal anti-NM2A (IB)	Biolegend	Cat# 909801 RRID: AB_291638
Rabbit polyclonal anti-NM2B (IB, IF)	Biolegend	Cat# 909901 RRID: AB_291639
Rabbit polyclonal anti-NM2C (IB)	Cell Signaling Technology	Cat# 8189 RRID: AB _10886923
Mouse monoclonal anti-pan-actin (IB)	Merck MilliporeSigma, Bedford, MA, USA	Cat# mab1501 RRID: AB_2223041
FITC-phalloidin (IF)	Merck MilliporeSigma, Bedford, MA, USA	Cat# P5282
Mouse monoclonal anti-β-tubulin (IB)	Thermo Fisher Scientific, Waltham MA USA	Cat# 32-2600 RRID: AB_2533072
Rat monoclonal anti-ZO-1 (IF)	Prof. Daniel Goodenough, Harvard Medical School	Cat# R40.76 RRID: AB_2205518
Mouse monoclonal anti-ZO-1 (IB)	Thermo Fisher Scientific, Waltham MA USA	Cat# 3391000 RRID: AB_2533147
Rabbit polyclonal anti-cingulin (IB, IF)	Citilab	C532
Mouse monoclonal anti-occludin (IF, IB)	Thermo Fisher Scientific, Waltham MA USA	Cat# 33-1500 RRID: AB_87033
Rabbit polyclonal anti-E-cadherin (IF)	Santa Cruz, Santa Cruz, CA, USA	Cat# 7870 RRID: AB_2076666
Mouse monoclonal anti-E-cadherin (IB)	BD Biosciences, San Jose, CA, USA	Cat# BD 610181 RRID: AB_397580
Cy3-AffiniPure Donkey anti-Mouse IgG	Jackson ImmunoResearch Laboratories, West Grove, PA, USA	Cat# 715-165-151 RRID: AB_2315777
Cy3-AffiniPure Donkey anti-Rat IgG	Jackson ImmunoResearch Laboratories, West Grove, PA, USA	Cat# 712-166-150 RRID: AB_2340668
Alexa Fluor 488-AffiniPure Donkey anti-Rabbit IgG	Jackson ImmunoResearch Laboratories, West Grove, PA, USA	Cat# 711-545-152 RRID: AB_2313584
Cy5-AffiniPure Donkey anti-Rat IgG	Jackson ImmunoResearch Laboratories, West Grove, PA, USA	Cat# 712-175-153 RRID: AB_2340672
Cy5-AffiniPure Donkey anti-Mouse IgG	Jackson ImmunoResearch Laboratories, West Grove, PA, USA	Cat# 715-175-150 RRID: AB_2340819
Anti-mouse IgG (H+L), HRP Conjugate	Promega , Madison, WI, USA	Cat# W4021 RRID: AB_430834
Anti-rabbit IgG (H+L), HRP Conjugate	Promega , Madison, WI, USA	Cat# W4011 RRID: AB_430833
**Plasmids**		
GFP-ZO-1	[43]	S2474
**Chemicals, Reagents, Peptides, Critical commercial assays**		
Pierce Protease Inhibitor Tablet, EDTA-free	Thermo Fisher Scientific, Waltham MA USA	Cat# A32965
jetOPTIMUS	Polyplus, Illkirch, France	Cat# 117-15

Lipofectamine RNAiMAX	Invitrogen, Waltham, MA, USA	Cat# 13778030
S-MEM	Gibco™ Thermo Fisher Scientific, Waltham MA USA	Cat# 11380-037
DMEM	PAN Biotech, Aidenbach, Germany	Cat# P04-04500
OptiMeM	Gibco™ Thermo Fisher Scientific, Waltham MA USA	Cat# 51985-026
Pierce BCA Protein assay kit	Thermo Fisher Scientific, Waltham MA USA	Cat# 23225
Nitrocellulose membrane	Roth, Arlesheim, Switzerland	Cat# 9302-1
WesternBright ECL kit	Advansta, Menlo Park, CA, USA	Cat# K-12045-D50
Molecular Weight Markers for SDS-PAGE	BioRad, Hercules, CA, USA	Cat# 1610373
DAPI-Hoechst nuclear dyes	Thermo Fisher Scientific, Waltham MA USA	Cat# 33342
Matrigel	BD Biosciences, San Jose, CA, USA	Cat# 354230
Epidermal Growth Factor (EGF)	Merck MilliporeSigma, Bedford, MA, USA	Cat# E-4127
Fluoromount-G	Thermo Fisher Scientific, Waltham MA USA	Cat# 00-4958-02
Round glass coverslips, 14 mm diameter	Thermo Fisher Scientific, Waltham MA USA	Cat# 50-189-7775
Glass-bottom culture dishes, 27 mm diameter	Thermo Fisher Scientific, Waltham MA USA	Cat# 150682
Cantilever MLCT C	Bruker AFM Probes, Camarillo, CA, USA	N/A
Dulbecco's Phosphate Buffered Saline (PBS)	Gibco™ Thermo Fisher Scientific, Waltham MA USA	Cat# 21600-051
Ethylenediaminetetraacetic acid (EDTA)	Invitrogen, Waltham, MA, USA	Cat# AM9260G
Fetal Bovine Serum	Pan Biotech, Aidenbach, Germany	Cat# P30-3306
Fetal Bovine Serum (FBS) Premium	Biowest, Nuaillé, France	Cat# S181B-500
HEPES-solution	Merck MilliporeSigma Bedford, MA, USA	Cat# H0887
MEM, GlutaMAX™ Supplement	Gibco™ Thermo Fisher Scientific, Waltham MA USA	Cat# 41090093
NEAA (Non Essential Amino Acids) Supplement	Gibco™ Thermo Fisher Scientific, Waltham MA USA	Cat# 11140050
Trypsin 2.5 %	Gibco™ Thermo Fisher Scientific, Waltham MA USA	Cat#15090-046
Penicillin-Streptomycin	Merck MilliporeSigma Bedford, MA, USA	Cat#P43333
Petri dish (μ-Dish, ibiTreat, 35 mm, low, polymer coverslip	Ibidi, Gräfelfing, Germany	Cat# 80136
**Experimental models: Cell lines**		
Madin-Darby Canine Kidney Tet-Off (MDCK) WT	A Fanning, University of North Carolina	Clontech, Takara
Madin-Darby Canine Kidney Tet-Off (MDCK) NM2B-KO	This paper	N/A
**Oligonucleotides**		
CRISPR target sequence: canis NM2B TTGCTTCTTCGCATAAAGG	This paper	Genscript
CRISPR target sequence: canis NM2B TTTGCAAGCAAATCCAATCC	This paper	Genscript
**Software and algorithms**		
Fiji/Image J, Version 2.16.0/1.54g	N/A	Imagej.nih.gov/ij/

		RRID: SCR_003070
Affinity Designer, Version 1.10.8	N/A	https://affinity.serif.com/<br>RRID: SCR_016952
Prism GraphPad, Version 10	N/A	https://www.graphpad.com/scientific-software/prism/<br>RRID: SCR_002798
Snapgene Version 3.1.2	N/A	snapgene.com<br>RRID:SCR_015052
Python, Version 3.10	N/A	Python.org<br>RRID:SCR_008394
