# Supplementary figures and images for: Nonmuscle Myosin-2B Regulates Apical Cortical Mechanics, ZO-1 Dynamics and Cell Size in MDCK Epithelial Cells
Source: Cells. 2025 Jul 23;14(15):1138. doi: 10.3390/cells14151138 (PMC12346661; doi:10.3390/cells14151138)

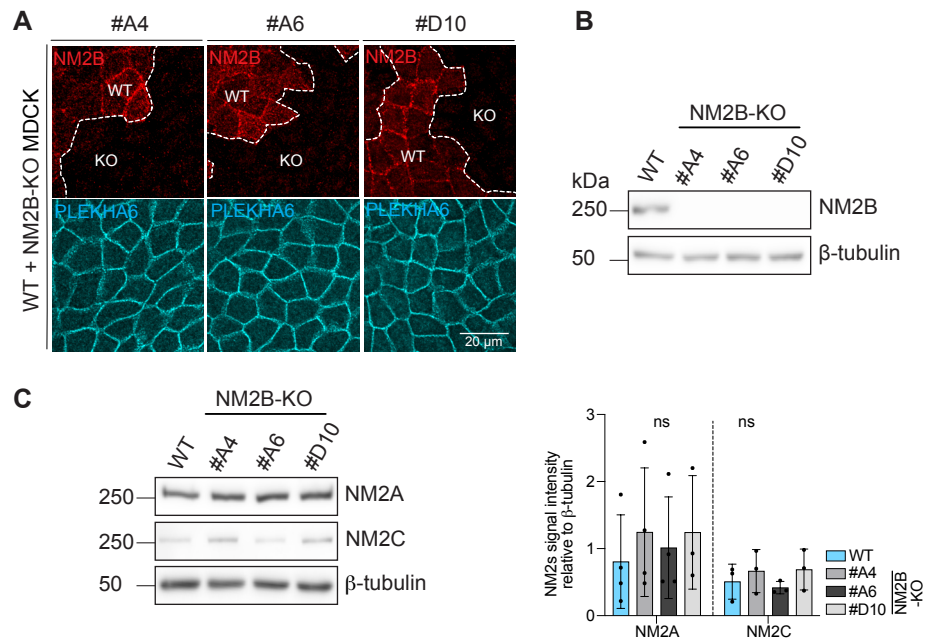

Supplementary Figure 1

Supplement: Supplementary file 1 [file cells-14-01138-s001.zip › Supplementary Figure 1.pdf]

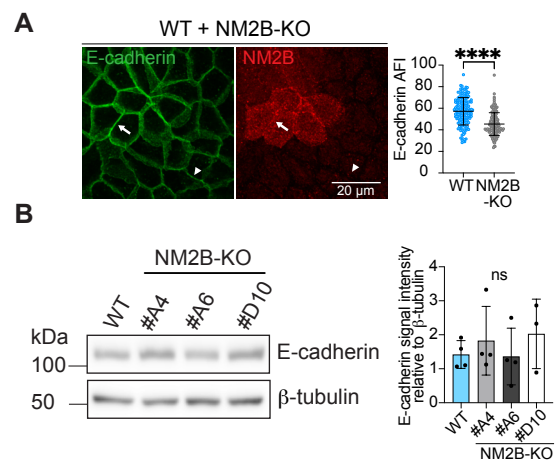

Supplementary Figure 2

Supplement: Supplementary file 1 [file cells-14-01138-s001.zip › Supplementary Figure 2.pdf]
